# Latent Growth Curve Modeling for COVID-19 Cases in Presence of Time-Variant Covariate

**DOI:** 10.1155/2022/3538866

**Published:** 2022-02-18

**Authors:** M. S. Panwar, C. P. Yadav, Harendra Singh, Taghreed M. Jawa, Neveen Sayed-Ahmed

**Affiliations:** ^1^Department of Statistics, Banaras Hindu University, Varanasi 221005, India; ^2^Department of Mathematics, Post Graduate College, Gazipur 233001, India; ^3^Department of Mathematics and Statistics, College of Science, Taif University, P. O. Box 11099, Taif 21944, Saudi Arabia

## Abstract

For the past two years, the entire world has been fighting against the COVID-19 pandemic. The rapid increase in COVID-19 cases can be attributed to several factors. Recent studies have revealed that changes in environmental temperature are associated with the growth of cases. In this study, we modeled the monthly growth rate of COVID-19 cases per million infected in 126 countries using various growth curves under structural equation modeling. Moreover, the environmental temperature has been introduced as a time-varying covariate to enhance the performance of the models. The parameters of growth curve models have been estimated, and accordingly, the results are discussed for the affected countries from August 2020 to July 2021.

## 1. Introduction

The coronavirus disease (COVID-19) was first reported in Wuhan city, China. Several individuals in Wuhan's seafood market were identified with unknown viral pneumonia [1–3]. In the next few months, the virus, severe acute respiratory syndrome coronavirus 2 (SARS-CoV-2), spread to other cities of China and then worldwide. On 30 January 2020, the Director-General of the World Health Organization (WHO) declared the outbreak of COVID-19 to be a public health emergency of international concern. In March 2020, more than one hundred countries were facing challenges due to this virus, and the infection cases from COVID-19 were identified almost all over the world. Since March 2020, there are now specific vaccines available against SARS-CoV-2. In the absence of specific therapeutic drugs or vaccines, controlling the spread of SARS-CoV-2 was nearly impossible, as the health management system of any country was not sufficient enough to deal with this pandemic [4–7].

According to the WHO reports [8], more than 214 million cases had been reported globally by the end of August 2021, out of which around 4.47 million deaths occurred. Here, it was noticed that the growth rate of infected cases and deaths experienced in different regions were dissimilar. A number of causes can affect the growth rate of cases in a region or country, such as the health management system, government policy, and environmental factors. At the initial phase, partial or complete lockdown and quarantine played an important role in controlling the spread of the virus. The work by Bacchetti et al., in [9], showed that lockdown was highly effective in reducing mortality in more polluted areas at the early stage of the pandemic. Moreover, Marquez et al. [10] concluded that air pollution results in a higher incidence and mortality from COVID-19. Azuma et al. studied the role of various environmental factors in the transmission of SARS-CoV-2 in indoor spaces [11].

In the first quarter of 2020, in a study of COVID-19 cases and the related meteorological factors in 122 cities of China, no evidence was found that the case counts of COVID-19 will decline when the weather becomes warmer [12]. On the contrary, an earlier study in the laboratory by Casanova et al. [13] has verified that SARS-CoV can be inactivated rapidly as temperature increases from 4°C to 40°C. Even though the data were quite limited for the second quarter of 2020, Mandal and Panwar [14] and Shao et al. [15] have suspected that the spread of SARS-CoV-2 may also be affected by the change in temperature. Thereafter, many researchers have established the association between the temperature and COVID-19 cases [16–18]. For specific geographical regions, the relationship among both in the presence of some other factors was investigated [19–22].

Modeling of respiratory diseases is always of high priority for researchers. Moreover, the outbreak of COVID-19 presented a new challenge for everyone to deal with this situation. In the last few months, various approaches have been utilized to fit the growth of COVID-19 cases over time. Balli, in [23], has proposed a time-series prediction model to obtain the disease curve and predict the pandemic trend using machine learning methods. For this purpose, linear regression, multilayer perception, random forest, and support vector machine learning methods are utilized. Furthermore, the susceptible-infected-recovered (SIR) model is a well-known and widely used method for respiratory diseases. The classic SIR model was updated by incorporating four new factors that are crucial in fitting the data of COVID-19 cases [24]. Several works have modified the SIR model in the same manner [25–27].

Using the generalized logistic and generalized Richards model, Wu et al. [28] have presented the fitting for COVID-19 cases in China; then, a similar exercise was performed for the 33 other countries, which were at a less advanced stage at that time. Moreover, several fractional-order dynamical models for the analysis of the virus spread were proposed [29–33]. Few researchers have attempted the model fitting of the dynamics of COVID-19 cases in the presence of environmental temperature. Shi et al. [34] have used the modified susceptible-exposed-infectious-recovered (M-SEIR) model by incorporating the temperature factor to simulate the COVID-19 outbreak dynamics in Wuhan. In other studies, they examined the associations between epidemiological parameters of the dynamics of new cases and temperature using an autoregressive integrated moving average (ARIMA) model [35]. Moreover, Shah et al. [36] have proposed a compartmental mathematical model for the transmission dynamics of the COVID-19 under the Caputo fractional-order derivative. The Hilbert-type inequalities play a major role in mathematics for pattern complex analysis, numerical analysis, and qualitative theory of differential equations and their implementation [37–39].

Generally, a time-series, cross-sectional, or longitudinal data-based approach is utilized when a response variable is observed with respect to time. These methods have suitability concerns and accordingly advantages and disadvantages. In this study, we use structural equation modeling (SEM) with longitudinal data. These models are generally known as latent curve or growth curve models (GCMs). The rest of the article is organized as follows. In Section 2, various facts have been explored using appropriate plots for cases per million (CPM) and temperature over the months. Then, in Section 3, we build various GCMs for all country data and select the most suitable one for further analysis. In Section 4, the temperature has been added as a time-varying covariate in the modeling to enhance the performance of the considered GCM. In Section 5, all results are discussed with their interpretation. Furthermore, the complete article has been summarized and concluded in Section 6.

## 2. Exploratory Data Analysis

In this study, the data for global COVID-19 cases have been obtained from https://ourworldindata.org. A total of 126 countries have been considered for cases recorded from August 2020 to July 2021. The CPM given in a month represents the number of cases recorded on the fifteenth day of that month. Accordingly, the monthly temperature is collected for the capitals of all considered countries from https://www.weather-atlas.com. The value representing temperature in a month is the average temperature in that month.

**Figure 1:**
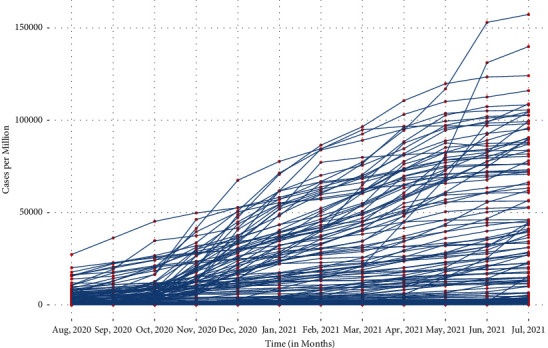
Trajectory plot for CPM over the months from August 2020 to July 2021.

Before starting the analysis, let us explore and discuss some hidden facts about the data. A simple monthly trajectory plot from August 2020 to July 2021 for all countries is given in Figure 1. In this duration, it is quite easy to observe that the growth of CPM in all countries is high in the first month and then stabilizes in most countries in the next few months. Nevertheless, many countries have experienced sudden rapid growth in CPM in the last few months of the year.

**Figure 2:**
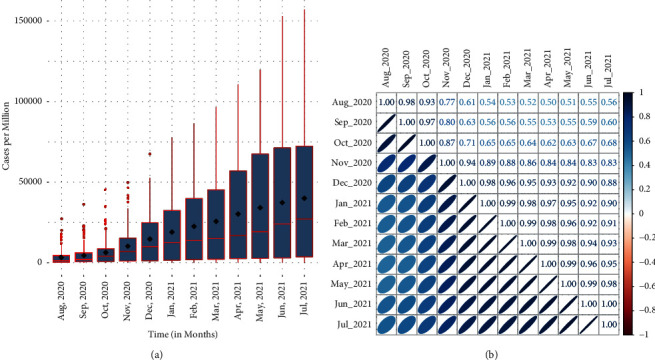
A set of box plot and correlation matrix plots of CPM from August 2020 to July 2021 for all countries. (a) Box plots for CPM over the months, (b) Correlation matrix plot for CPM over the months.

Figure 2(a) shows a set of box plots to understand the nature of the data over the months. The box plots in this figure show the CPM distribution over the months. It can be seen that the median and mean of CPM increase over the months and the mean is significantly larger than the median in all months. Thus, the distribution is positively skewed in all months. Moreover, the median and dispersion increase at a large scale over the months. In a few countries, CPM is very high, so these countries act as outliers in the first few months; however, in the last months, almost all match with the nature of the sample. A correlation matrix plot of CPM over the months is also shown in Figure 2(b). Except for the first three months, the correlation is high for months close together in time, but the correlation tends to decrease with increase in the time separation between the measurement months. On the contrary, in the first few months, the correlation decreased for the upcoming months but again started to increase. This is weak evidence; however, it is the very first indication that seasons may correlate with the growth of CPM. Moreover, a few basic statistics to understand the characteristics of the observed data are given in Table 1.

**Figure 3:**
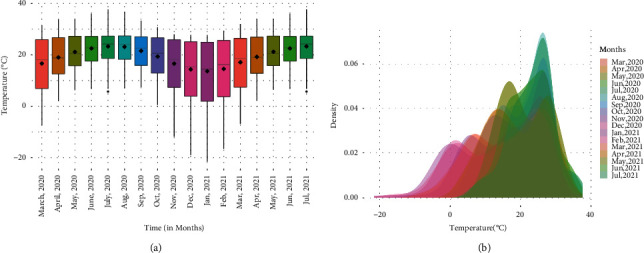
A set of box plots and density plots of temperature from August 2020 to July 2021 for all countries. (a)Box plot for temperature. (b)Density plot for temperature.

The distribution of global temperature over the months is given in Figure 3. It can be seen from Figure 3(a) that the global temperature distribution shifts upwards in the first half of the year and then in the second half it goes downwards. However, it does not mean that all the countries follow the same pattern. This can be observed from Figure 3(b) in which, for each month, a density plot has been sketched. The density plots are multimodal because over the months each country contributes to temperature distribution according to its position on the global map. In general, as latitude increases the temperature decreases. This latitude-wise varying pattern of temperature may have a significant impact on the growth of CPM for respective countries.

## 3. Model Building and Elicitation

The traditional methods for studying the changes in the linear and nonlinear framework are regression and analysis of variance (ANOVA). These approaches basically deal with the mean level differences, and among individuals, changes are observed from residuals. To utilize the information from residuals several methods such as random effect ANOVA, multilevel modeling and hierarchical linear modeling have been proposed. These models explore the differences among individuals with the help of random coefficients. However, the limitation of such models is that they are based on a single response variable. A single response variable is not able to capture all the complexities of a growth model (see [40]).

As the objective of this study is to observe the intraindividual changes and interindividual differences for all countries over the considered time period, so, to possess such characteristics, a structural equation modeling for longitudinal data has been proposed. GCMs, which are generally applied for modeling in social and behavioral sciences, are used for studying such changes. For GCM, response variables are observed over the ordered time periods whereas some time-invariant or time-variant covariates may also be present. The basics of GCM and hypothesis testing for individual change and interindividual differences are discussed and derived in [41]. In [42], authors have discussed GCM for different models and analyzed the cortisol production data. For more details about longitudinal studies using growth curve models, one may refer to [43–46]. For understanding the models based on latent variables with respective *R* code, one may follow [47].

As the primary objective of this study is to find a better model for CPM trajectory, as shown in Figure 1, so, in this section, some possible GCMs such as linear, exponential, latent, and multiphase have been introduced which could provide a better substitute for CPM fitting. The general structure of the GCM can be given as

(1) $$\begin{array}{c} C{\left[t\right]}_{n}=\Lambda_{0}\left[t\right].T_{0n}+\Lambda k\left[t\right].T_{\text{kn}}+\varepsilon {\left[t\right]}_{n}, \end{array}$$

where [*t*]_*n*_ is a multioccasion vector which represents the observed value of CPM for *n*^*th*^ country for *t*^*th*^ month. The set of vectors Λ_0_*, Λ*_1_*, , Λ*_*k*_ is collectively responsible for the intraindividual change, i.e., each of these captures the growth of CPM in a country over the months. This vector defines the shape of the interindividual change such as linear and exponential for a country. The latent or unobserved variables which are denoted by *τ*_0_*, τ*_1_*, , τ*_*k*_ define interindividual differences in intraindividual change among countries. In the defined model by (1), each interindividual difference variable *τ*_0_*, τ*_1_,…*, τ*_*k*_ is associated with the corresponding intraindividual change variable Λ_0_*, Λ*_1_,…*, Λ*_*k*_.

Generally, the set of latent variables *τ*_0_*, τ*_1_,…*, τ*_*k*_ has a multivariate normal distribution with mean vectors (*µ*_0_*, µ*_1_,…*, µ*_*k*_) and random variances and covariances *σ*_*ij*_; *i, j* = 1, 2,… *, k*. The mean vector captures the pattern of intraindividual change and variances, and covariances represent the extent to which countries differ within and between. The time-dependent residual variable, *ε*[*t*]_*n*_, is assumed to have a mean 0 and the same variance, *σ*^2^, at each occasion; also, it is assumed to be uncorrelated with other variables.

Let us first describe and derive some GCMs using (1) and then choose the best-fitted model for CPM among them. Few appropriate GCMs which are considered for trajectory fitting are linear, exponential, latent, and multiphase.

### 3.1. Linear Growth Curve Model

In a linear GCM, the growth of the outcome variable is in the form of a straight line which may be in a positive, negative, or constant direction over the time periods. A linear GCM can be described by two vectors, Λ_0_ and Λ_1_, for different countries over the months from model in (1). As the model is applied from August 2020 to July 2021, hence, we have

(2) $$\begin{array}{c} \Lambda_{0}\left[t\right]=\left[1,1,1,1,1,1,1,1,1,1,1,1\right],\\ \Lambda_{1}\left[t\right]=\left[0,1,2,3,4,5,6,7,8,9,10,1\right], \end{array}$$

where Λ_0_ is used to describe the initial level of measurement of the outcome variable when the other effects are 0, whereas Λ_1_ is responsible for the growth or decline in C_n_. This means that the countries can differ from each other in two ways such as their latent intercept (*τ*_0_) and latent slope (*τ*_1_). All entries for Λ_0_ are fixed to 1, this means that intercept affects all measures with equal scores across months.

### 3.2. Quadratic Growth Curve Model

In general, the changes over time in measurement variable are nonlinear. So, we want to introduce more complexity in the model to capture this nonlinearity. For this purpose, we are introducing another vector Λ_2_, which is responsible for quadratic change in the intraindividual change and interindividual differences. Here, another two vectors Λ_0_ and Λ_1_ are responsible for the intercept and linear change in trajectory. We define three vectors as Λ_0_[*t*] = [1, 1, 1, 1, 1, 1, 1, 1, 1, 1, 1, 1], Λ_1_[*t*] = [0, 1, 2, 3, 4, 5, 6, 7, 8, 9, 10, 1], and Λ_2_[*t*] = (Λ_1_[*t*])^2^ = [0, 1, 4, 9, 16, 25, 36, 49, 64, 81, 100, 1].

The initial level amount of outcome variable is depicted by *τ*_0_, and after then, at each successive time periods, the linear and quadratic changes are governed by vectors *τ*_1_ and *τ*_2_.

### 3.3. Exponential Growth Curve Model

In exponential GCM, the two vectors, Λ_0_ and Λ_1_, are responsible for the exponential intraindividual change, and these can be defined such as

(3) $$\begin{array}{c} \Lambda_{0}\left[t\right]=\left[1,1,1,1,1,1,1,1,1,1,1,1\right], \end{array}$$

where *λ* can be estimated from the observations. The interindividual change among the countries is depicted by two latent random variables *τ*_0_ and *τ*_1_. Here, the random variable *τ*_0*n*_ can be interpreted as the maximum level of *C*_*n*_. The sum of latent slope score and latent asymptotic score (*τ*_0*n*_ + *τ*_1*n*_) represents the value of *C*_*n*_[0]. The random variable *τ*_1*n*_ represents a country's potential for change in _*n*_[*t*] from initial level to upcoming months. The parameter *λ* indicates the rate at which the level of *C*_*n*_[*t*] changed to the asymptotic level and here is modeled as being identical for all countries, meaning that the rate at which any individual's _*n*_[*t*] level changes is unidirectional (either continuously increasing or decreasing toward his or her asymptotic capacity, *τ*_0*n*_) and constant (exponentially) across the entire observation period from August 2020 to July 2021. This assumption can be relaxed in further studies.

### 3.4. Latent Growth Curve Model

The basis coefficients for a latent GCM are estimated freely so that the optimal change in trajectory can be achieved as per the nature of data, whereas, in earlier discussed models, the basis coefficients have been fixed in advance. Here, the basis coefficients are defined such as

(4) $$\begin{array}{c} \Lambda_{0}\left[t\right]=\left[1,1,1,1,1,1,1,1,1,1,1,1\right],\\ \Lambda_{1}\left[t\right]=\left[0,\lambda_{1},\lambda_{2},\lambda_{3},\lambda_{4},\lambda_{5},\lambda_{6},\lambda_{7},\lambda_{8},\lambda_{9},\lambda_{10},1\right]. \end{array}$$

We fixed the first and last basis coefficients as 0 and 1, as it is necessary for the model identification. In the case of latent GCM, the nonlinear pattern of intraindividual change is captured by vector Λ_1_[*t*] and a single interindividual difference variable *τ*_1_.

### 3.5. Multiphase Growth Curve Model

A multiphase GCM is based on different spline regression models that are connected for different time slots. As it is observed that various countries are facing a number of COVID-19 waves, so a multiphase model may be a good choice for CPM modeling. Figure 1 shows such pattern where the rate of change of COVID-19 cases is not uniform over considered months. From many possible multiphase GCMs, particularly, MP_[3,4,5]_, has been taken for modeling. The suffix vector specifies the length of phases considered in the model. As the data are taken from August 2020 to July 2021, hence, the vector [7, 25, 37] denotes three phases are taken as Phase I (August 2020, September 2020, and October 2020), Phase II (November 2020, December 2020, January 2021, and February 2021), and Phase III (March 2021, April 2021, May 2021, June 2021, and July 2021). In the MP_[3,4,5]_ model, Phase I is known as baseline phase and modeled via Λ_0_, whereas Phase II and Phase III are modeled via Λ_1_ and Λ_2_, respectively. Accordingly, the three intraindividual change vectors become

(5) $$\begin{array}{c} \Lambda_{0}\left[t\right]=\left[1,1,1,1,1,1,1,1,1,1,1,1\right],\\ \Lambda_{1}\left[t\right]=\left[0,0,0,\lambda_{3},\lambda_{4},\lambda_{5},1,1,1,1,1,1,1\right],\\ \Lambda_{2}\left[t\right]=\left[0,0,0,0,0,0,0,\lambda_{7},\lambda_{8},\lambda_{9},\lambda_{10},1\right], \end{array}$$

where the values of *λ*_*js*_ can be estimated from the data.

The interindividual difference is governed by the latent random variables *τ*_0_, *τ*_1_, and *τ*_2_. The means of the latent variables *τ*_0_, *τ*_1_, and *τ*_2_ represent the average baseline *C*[*t*]_*n*_ level, amount of [*t*]_*n*_ change in second phase, and amount by which [*t*]_*n*_ gains in the last phase, respectively. Simultaneously, the variances of the latent variables represent the extent to which countries differ in these aspects of intraindividual change and how interindividual differences in one aspect are related to interindividual differences in the other aspects which can be defined by their covariances.

**Figure 4:**
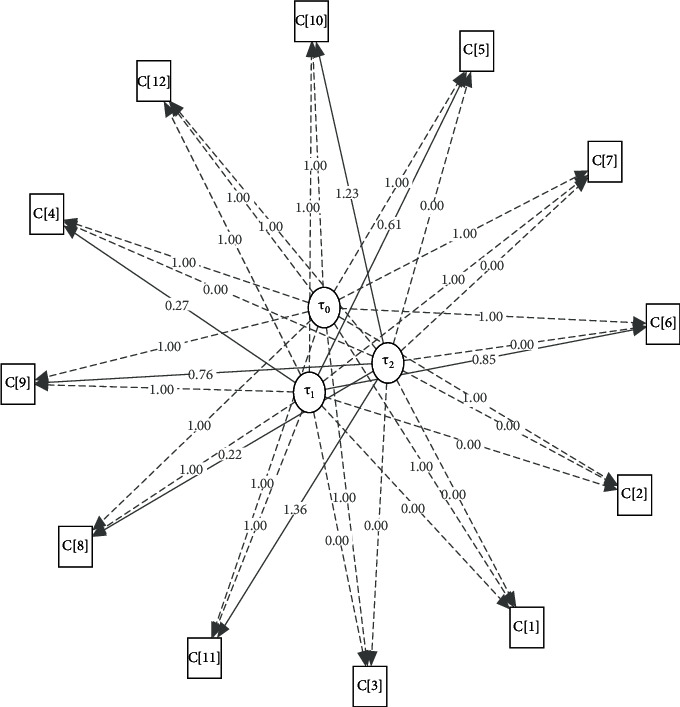
Structure plot for MP_[3,4,5]_.

To find the best among the considered models, a few well-known model fitting criteria, such as Akaike Information Criterion (AIC), Bayesian Information Criterion (BIC), Tucker–Lewis Index (TLI), Root Mean Square Error of Approximation (RMSEA), and *χ*^2^-statistic with degrees of freedom (D.F.), have been taken. Lower values of AIC, BIC, RMSEA, and *χ*^2^-statistic and higher values of TLI statistic indicate the choice of a suitable model. Based on these criteria, it can be seen from Table 2 that the multiphase model, MP_[3,4,5]_, is performing better than others. So, it can be concluded that multiphase GCM is most appropriate among the considered models. The estimates of the coefficients for all considered GCMs are given in Table 3 and discussed in Section 5. For the multiphase GCM, the structure plot is given in Figure 4. In the structure plot, the dotted lines show the fixed factor loading and the dark line shows the estimated elements of factor loading. It is expected that, with the addition of suitable covariates, the overall performance of the multiphase GCM may also improve.

## 4. Modeling with Time-Varying Covariate

Many factors are influencing the growth of CPM, but the environmental temperature has its own significant impact. Earlier, in Section 2, it has been shown that there may be some dependency between CPM and temperature. Also, from Figures 2(a) and 3(a), it can be seen that the monthly gain in CPM depends on the pattern of global temperature. If the temperature is added as a covariate in all country data, then the GCMs will perform robustly.

**Figure 5:**
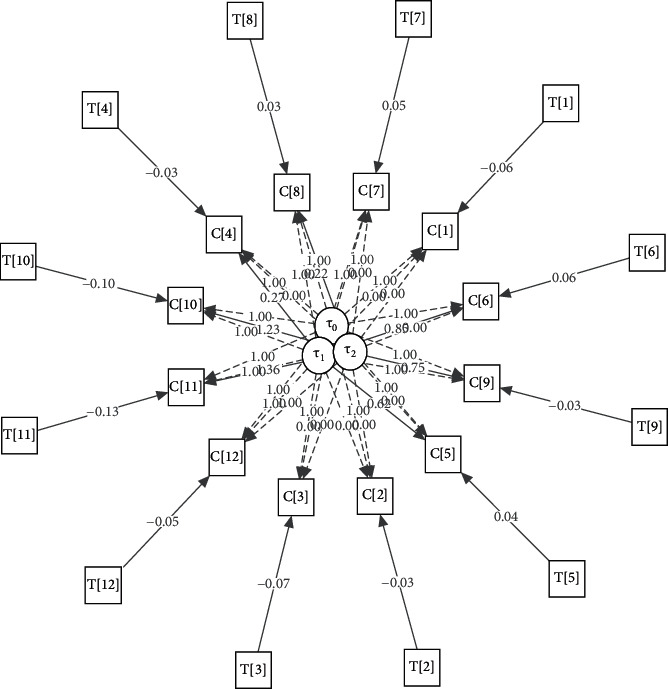
Structure plot for MP_[3,4,5]_^*T*^.

In general, two types of predictor variables are used in longitudinal studies. The covariate is constant over the measurement time called time-invariant covariate (TIC) and is varying over time periods called time-varying covariate (TVC). Here, in this study, the temperature is a TVC over the months, which have a direct impact on the CPM with some coefficient, say, *γ*[*t*]. Now, the model defined by (1) can be redefined in the presence of TVC as follows:

(6) $$\begin{array}{c} C{\left[t\right]}_{n}=\Lambda_{0}\left[t\right].T_{0n}+\Lambda_{1}\left[t\right].T_{1n}+\cdots +\Lambda_{k}\left[t\right].T_{\text{kn}}+Y\left[t\right]T{\left[t\right]}_{n}+\varepsilon {\left[t\right]}_{n}, \end{array}$$

where *T* [*t*]_*n*_ represents the temperature of *n*^*th*^ country in the *t*^*th*^ month. All GCMs in presence of TVC are performing better than the respective models introduced in the previous section. Also, we tried different possible combinations of phases to construct multiphase models with TVC. Among the considered models, MP_[3,4,5]_^*T*^ is outperforming with AIC, BIC, TLI, RMSEA, and *χ*^2^-statistic (d.f) values 18722.02, 18843.50, 0.79, 0.07, and 361.44(194), respectively. In Table 3, the estimate of coefficients for all considered GCMs with TVC has been given. For the multiphase GCM MP_[3,4,5]_^*T*^, the structure plot is given in Figure 5. In the structure plot, the dotted lines show the fixed factor loadings and the dark lines represent the estimated elements of factor loadings and estimated coefficients for TVCs.

## 5. Results

In this section, we are discussing about the results from Tables 3 and 4. In Table 3, the estimate of parameters of four GCMs has been given. For linear GCM, the mean baseline level of *C*[*t*]_*n*_ is 77.1223 (*τ*_0_) and then growth is measured with an increment of 0.0554 (*τ*_1_) over the months. In quadratic GCM, the mean baseline level of the outcome variable is 77.5197 and amount of suppressing and increment in linear and quadratic phases is 0.3052 and 0.0240 respectively. In exponential GCM, the baseline level is 76.9430 which is the sum of *τ*_0_ and *τ*_1_. After this, growth in [*t*]_*n*_ is observed with an exponential rate of 15.9286 to some limit or capacity of a country. The average baseline value from latent GCM is 77.2063 and the average total amount of growth is *−*0.2633. The estimated value of *C*[*t*]_*n*_ at any month can be calculated by [77.2063 + (*−*0.2633)*λ*_*t*_], e.g., in Oct 2020 the estimated value of *C*[*t*]_*n*_ is 76.99637. For the considered multiphase model, *MP*_[3,4,5]_, the average baseline value is 77.0714. The average growth amount from November 2020 to February 2021 is −0.5005, and the additional growth amount is 0.2096 from March 2021 to July 2021.

Similar to latent GCM, one can estimate *C*[*t*]_*n*_ using the estimates of respective coefficients for the multiphase model also. The covariances for *τ*_0_*, τ*_1_ and *τ*_2_ for all models are also provided in the table. The variance terms represent the extent to which countries differ at the initial level in intraindividual change and the covariances indicate interindividual differences.

In Table 4, estimates of coefficients are observed for various GCMs in presence of TVC, temperature. In this table, all coefficients can be interpreted in a similar manner as in Table 3, except regression coefficients due to covariate. The regression coefficients can be defined as one unit change in temperature at time *t* which is associated with *γ*[*t*] unit change in [*t*]. Here, it is noticeable that, almost in all months, temperature is negatively associated with the growth of CPM.

## 6. Conclusion

In this study, growth in CPM due to COVID-19 is considered a variable of interest. For different countries, the trajectories of CPM are studied from August 2020 to July 2021. The intraindividual change and interindividual differences were captured using linear, exponential, latent, and multiphase GCMs. Based on certain criteria, the multiphase GCM performs better than the other models. Therefore, it can be preferred for analysis purposes. A number of factors are responsible for the rapid growth of CPM in a country. Moreover, these factors impact different countries with different weights. Thus, in this study, environmental temperature is considered a covariate that significantly impacts the growth of CPM. Different GCMs were fitted to the data without and with a covariate. Based on various fitting criteria, it is noticed that GCMs improve when the temperature is introduced as a covariate. So, we can say that temperature may be one of the reasons responsible for the changes in CPM over the months. Nevertheless, other possible factors may have an important role in the growth of CPM and can be included in the model for further study. The inclusion of other factors in models may improve results. Furthermore, for the study of growth in CPM for a particular region, there may be differences in the model-based outcomes.

## Figures and Tables

**Table 1:** Basic statistical measures for CPM over the months.

	Aug 2020	Sep 2020	Oct 2020	Nov 2020	Dec 2020	Jan 2021	Feb 2021	Mar 2021	Apr 2021	May 2021	June 2021	July 2021
Mean	3461.19	4601.82	6300.93	10244.54	14673.75	18986.02	22494.01	25658.11	30217.82	34152.76	37279.40	39993.10

Median	1336.83	2137.14	3972.78	6961.24	9917.47	12441.64	13814.11	15031.02	16929.84	19220.02	24037.40	27130.06

Standard deviation (S.D.).	4761.05	6104.34	7820.51	11311.03	15723.94	20345.31	23985.04	26961.99	31248.33	34687.31	37657.38	39044.99

Range	27279.48	36215.94	45213.40	49765.78	67480.17	77670.9	86453.79	96455.48	110589.5	119737.5	152931.6	157212.4

**Table 2:** Different fitting criteria for various GCMs of all countries data from August 2020 to July 2021.

Model	AIC	BIC	TLI	RMSEA	*χ* ^2^-statistic (D.F.)
Linear	18993.40	19045.03	0.47	0.19	497.96 (73)
Quadratic	18790.03	18853.81	0.71	0.14	286.59 (69)
Exponential	19093.62	19148.29	0.34	0.22	596.19 (72)
Latent	18916.38	18998.37	0.51	0.19	400.94 (63)
Multiphase	18702.93	18787.96	0.82	0.11	185.49 (62)

**Table 3:** Estimate of coefficients for various GCMs for all countries data from August 2020 to July 2021.

Intercept and slope:					
	Linear	Quadratic	Exponential	Latent	Multiphase
*τ*_0_	77.1223^*∗*^	77.5197^*∗*^	77.5291^*∗*^	77.2063^*∗*^	77.0714^*∗*^
*τ*_1_	−0.0554	−0.3052	−0.0486	−0.2633	−0.5005^*∗*^
*τ*_2_		0.0240			0.2096^*∗*^
*λ* (rate)			15.9286^*∗*^		
*λ*_1_				−0.1469	
*λ*_2_				0.3803	
*λ*_3_				0.7973^*∗*^	0.2749^*∗*^
*λ*_4_				1.6157^*∗*^	0.0705^*∗*^
*λ*_5_				2.1637^*∗*^	0.8472^*∗*^
*λ*_6_				2.4558^*∗*^	
*λ*_7_				2.4272^*∗*^	0.2223^*∗*^
*λ*_8_				1.9969^*∗*^	0.7587^*∗*^
*λ*_9_				1.5036^*∗*^	1.2315^*∗*^
*λ*_10_				1.1624^*∗*^	1.3624^*∗*^

Covariances:					
*τ*_0_ *↔ τ*_0_	231.1603^*∗*^	634.8185^*∗*^	−7.1813	322.9.86^*∗*^	354.4765^*∗*^
*τ*_1_ *↔ τ*_1_	22.4033^*∗*^	383.8042^*∗*^	4.2484	402.1072	2508.8822^*∗*^
*τ*_2_ *↔τ*_2_	1428.2694^*∗*^	3.4922^*∗*^			
*τ*_0_ *↔ τ*_1_	−446.6204^*∗*^	−18.0949	−268.0263	−605.1945^*∗*^	−46.4837^*∗*^
*τ*_0_ *↔ τ*_2_	39.8306^*∗*^	35.6665^*∗*^			
*τ*_1_ *↔ τ*_2_		−35.5733^*∗*^			−1029.3199^*∗*^

^*∗*^ denote significant parameter at *p* < 0.05.

**Table 4:** Estimate of coefficients for various GCMs with TVC for all countries data from August 2020 to July 2021.

	Linear	Quadratic	Exponential	Latent	Multiphase
Intercept and slope					
*τ*_0_	75.7481^*∗*^	86.0991^*∗*^	88.0494^*∗*^	82.0764^*∗*^	79.8183^*∗*^
*τ*_1_	0.1262	−5.3411	−1.2446^*∗*^	−2.4983^*∗*^	−6.8267
*τ*_2_		0.5547^*∗*^			7.8347
*λ* (rate)			9.8281		
*λ*_1_				−0.1288	
*λ*_2_				0.4034	
*λ*_3_				0.8180^*∗*^	0.2743^*∗*^
*λ*_4_				1.6236^*∗*^	0.6174^*∗*^
*λ*_5_				2.1601^*∗*^	0.0597^*∗*^
*λ*_6_				2.4385^*∗*^	
*λ*_7_				2.4080^*∗*^	0.2245^*∗*^
*λ*_8_				1.9792^*∗*^	0.7487^*∗*^
*λ*_9_				1.4700^*∗*^	1.2254^*∗*^
*λ*_10_				1.1387^*∗*^	1.3616^*∗*^

Covariances					
*τ*_0_ *↔ τ*_0_	233.4468^*∗*^	625.8157^*∗*^	−9.0008	313.6485^*∗*^	348.3898^*∗*^
*τ*_1_ *↔ τ*_1_	22.3028^*∗*^	385.5562^*∗*^	12.2186^*∗*^	411.1636	2485.5481^*∗*^
*τ*_2_ *↔τ*_2_		3.5006^*∗*^		1414.3588^*∗*^	
*τ*_0_ *↔ τ*_1_	−46.4866^*∗*^	−445.1598^*∗*^	−34.4772	−272.0185	−596.6951^*∗*^
*τ*_0_ *↔ τ*_2_	39.4834^*∗*^	21.3108			
*τ*_1_ *↔ τ*_2_		−35.7064^*∗*^			−1014.6153^*∗*^

Regression (TVC)					
*γ*[1]	0.0018	−0.1782	−0.1836	−0.0994	−0.0605
*γ*[2]	0.0316	−0.0524	0.0614	−0.0715	−0.0338
*γ*[3]	−0.0002	−0.0289	0.0131	−0.0719	0.0652
*γ*[4]	−0.0132	−0.0168	−0.0256	−0.0632	0.0334
*γ*[5]	0.0334	0.0589	0.0363	0.0052	0.0381
*γ*[6]	0.0314	0.0643	0.0376	0.0178	0.0561
*γ*[7]	0.0191	0.0373	0.0251	0.0077	0.0501
*γ*[8]	0.0276	0.0215	0.0371	0.0130	0.0340
*γ*[9]	−0.0005	−0.0346	0.0087	−0.0181	−0.0277
*γ*[10]	−0.0228	−0.1161^*∗*^	−0.0233	−0.0690	−0.1033
*γ*[11]	−0.0162	−0.2055^*∗*^	−0.0138	−0.0757	−0.1268
*γ*[12]	0.0233	−0.0570	0.0256	−0.0318	−0.0539

^*∗*^ denotes significant parameter at *p* < 0.05.

## Data Availability

The data used to support the findings of the study are obtained from the corresponding author upon request.
